# Estimation of pathogenic potential of an environmental *Pseudomonas aeruginosa* isolate using comparative genomics

**DOI:** 10.1038/s41598-020-80592-8

**Published:** 2021-01-14

**Authors:** Carola Berger, Christian Rückert, Jochen Blom, Korneel Rabaey, Jörn Kalinowski, Miriam A. Rosenbaum

**Affiliations:** 1 Bio Pilot Plant, Leibniz Institute for Natural Product Research and Infection Biology, Hans-Knöll-Institute (HKI), Beutenbergstr. 11a, 07745 Jena, Germany; 2 Center for Biotechnology - CeBiTec, University of Bielefeld, Bielefeld, Germany; 3 Bioinformatics and Systems Biology, Justus-Liebig University Gießen, Giessen, Germany; 4 Laboratory of Microbial Ecology and Technology (LabMET), Ghent University, Ghent, Belgium; 5 Faculty of Biological Sciences, Friedrich Schiller University, Jena, Germany

**Keywords:** Classification and taxonomy, Genome informatics, Bacterial genomics, Pathogens

## Abstract

The isolation and sequencing of new strains of *Pseudomonas aeruginosa* created an extensive dataset of closed genomes. Many of the publicly available genomes are only used in their original publication while additional in silico information, based on comparison to previously published genomes, is not being explored. In this study, we defined and investigated the genome of the environmental isolate *P. aeruginosa* KRP1 and compared it to more than 100 publicly available closed *P. aeruginosa* genomes. By using different genomic island prediction programs, we could identify a total of 17 genomic islands and 8 genomic islets, marking the majority of the accessory genome that covers ~ 12% of the total genome. Based on intra-strain comparisons, we are able to predict the pathogenic potential of this environmental isolate. It shares a substantial amount of genomic information with the highly virulent PSE9 and LESB58 strains. For both of these, the increased virulence has been directly linked to their accessory genome before. Hence, the integrated use of previously published data can help to minimize expensive and time consuming wetlab work to determine the pathogenetic potential.

## Introduction

*Pseudomonas aeruginosa* has been isolated from terrestrial and marine soil, fresh and salt water, sewage, plants, animals, and humans^1^. For the latter habitats, it is known as an opportunistic pathogen, which usually spreads to already vulnerable patients, causing ~ 10% of all nosocomial infections in most European Union hospitals^2^. Its combinatory virulence is transmitted through the action of a myriad of virulence factors. Not every *P. aeruginosa* isolate conveys an equal level of virulence to a given infection model and a strain that is effective in infecting a plant does not necessarily show an equal amount of virulence towards an animal^3,4^. For the frequently researched *P. aeruginosa* PA14 strain this increased virulence, as compared to the type strain PAO1, is mainly due to the presence of additional virulence factors. Their genes are predominantly clustered on two genomic islands (GIs) termed *P. aeruginosa* pathogenicity islands (PAPI)^5^.

Due to short generation times, mutations are frequently observed in bacterial genomes, which makes them a dynamic rather than a static gene collection^6^. For *P. aeruginosa,* numerous studies have proven that the pan genome can be viewed as a mosaic of a conserved core (~ 90% of a specific genome) and variable accessory Sets. ^7–9^. Core genes are defined as genes with orthologues in nearly all strains, which show a conserved synteny and a low average nucleotide substitution rate^7^. One study suggests the core genome of the *P. aeruginosa* species, which makes up the smallest fraction of the pan genome, to consist of 4000–5000 open reading frames (ORFs)^4^. The second fraction is the accessory genome with about 10,000 genes. It can be grouped according to general features like the means of inter- and intrachromosomal relocation. By assigning different functional modules, it can be sorted into (i) integrative and conjugative elements (ICEs), (ii) replacement islands, (iii) prophages and phage-like elements, and (iv) transposons, insertion sequences (ISs) and integrons^7^. These genes are only shared by certain, but not all strains of the species and are mainly located in GIs and genomic islets (GIts). By definition, GIs have a size of at least 10 kb, while GIts are smaller than 10 kb. Both types of elements have been acquired via horizontal gene transfer^7^. They are the cause for alterations in the genome size of *P. aeruginosa*, which has been reported to range from 5.2 to 7.4 Mb^4,8^. By prokaryotic standards, this is considered rather big, encoding genes from numerous and distinct gene families. This highlights the great genetic and functional diversity of this species^7^. Depending on the encoded genes, GIs can be classified into four functional categories: (i) pathogenicity islands (PIs; predominantly encoding pathogenicity factors), (ii) resistance islands (RIs; predominantly encoding resistance functions), (iii) metabolic islands (MIs; predominantly encoding biosynthesis of (secondary) metabolites), and (iv) symbiotic islands (SIs; predominantly encoding genes related to a host-bacterium symbiotic relationship)^19^. The by far largest fractions of the pan genome are singletons and rare genes that are only shared by very few strains. Their estimated number is at least 30,000 for the *P. aeruginosa* species^4^.

Over the years, a different nomenclature was established naming the islands PAPI-X (*P. aeruginosa* pathogenicity island), PAGI-X (*P. aeruginosa* genomic island) and LESGI-X (Liverpool Epidemic Strain genomic island). It is important to note that no direct correlation between PAGI and LESGI exists and that the respective islands are not exclusive to the PA or LES strains of *P. aeruginosa*. Besides PAPI-I and PAPI-II of *P. aeruginosa* PA14, 42 other GI have been previously described in the *P. aeruginosa* species^9–12^, of which multiple have been directly linked to an increased pathogenicity of the harboring strains^12–15^. Different detection software packages are available to help identifying regions of foreign DNA within a given genome. As the algorithms use different characteristics, have a different degree of sensitivity, and different shortcomings, usually not one program is able to identify all GIs and GIts. Hence, a combination of multiple complementary tools should be applied to get a thorough detection. Here, we used the established SIGI-HMM^16^, IslandPath-DIMOB^17^, PHASTER^18^ and GIPSy^19^ bioinformatic tools.

In this study, we describe how the abundantly available sequencing information of a species like *P. aeruginosa* can be used to characterize a newly sequenced strain. To this end, we sequenced the KRP1 environmental isolate and characterized its phylogenetic relationship by using more than 100 previously published closed *P. aeruginosa* genomes. We further employed different GI detection software programs and manual mining, to investigate the genome composition of this exemplary strain. The strain KRP1 was first isolated from a microbial fuel cell as one of the dominating bacterial species responsible for the high electron transfer efficiency of the mixed community^20^. Our previous study has shown that this strain shows a remarkably different behavior in lab operated bioelectrochemical systems, as compared to other *P. aeruginosa* variants^21^, including an increased production of the redox-active pathogenicity factors phenazines. For deeper investigations into the reasons behind this phenomenon in the future, knowledge of the genomic make-up of this strain is needed. By comparing the genomic content with other highly virulent *P. aeruginosa* variants, we are able to make educated predictions of the strains pathogenetic potential, without having to perform time- as well as money consuming animal experiments. While these findings are only predictions and may not be considered proven until actual wet lab testing was performed, they can still be of substantial aid for the *Pseudomonas* community and the labs working with the strain in question.

## Results and discussion

### *Pseudomonas sp*. KRP1 belongs to the *P. aeruginosa* species

The in silico hybrid approach assembly of the de novo sequenced KRP1 strain resulted in two circular contigs of 6,162,740 bps and 575,136 bps. As a recent study points out, the choice of the assembly algorithm can have a profound impact on all subsequent analysis^22^. We therefore employed a combination of a short and long read assembler, followed by a manual curation to ensure fulfillment of the suggested 3 C criteria (contiguity, correctness and completeness)^22^. Synteny comparisons between this initial in silico assembly and closely related *P. aeruginosa* strains showed multiple rearrangements of the ORFs encoded on the putative mega plasmid. In *P. aeruginosa* PA14, the corresponding sequence is located between two large homologous ribosomal RNA clusters. These clusters are known to be spots of inner genome rearrangements within the *P. aeruginosa* species^3,23^. Therefore, PCR was used to investigate the DNA sequence surrounding the ribosomal RNA clusters on the main chromosome and on the potential mega plasmid. This resulted in a redefined genome structure of KRP1, with one circular chromosome, containing 6,301 annotated protein-coding genes (Table 1).

**Table 1:** Genomic overview of different *P. aeruginosa* strains used in this study. ANI analysis was performed with the EDGAR platform^24,25^

*P. aeruginosa* strain	Total length (bps)	G + C-content (%)	Number of predicted genes	ANI with KRP1 (%)	Comment	References
KRP1	6,737,396	66.3	6301			This study
PAO1	6,264,404	66.6	5700	99.24	Type strain	^23^
PA14	6,537,648	66.3	6177	98.36	Common research strain	^3^
LESB58	6,601,757	66.3	6135	98.81	Hyper virulent strain	^15^
FA-HZ1	6,866,790	66.2	6389	99.98	Closest sequenced relative to KRP1	^27^
W45909	6,777,566	66.2	6475	99.96	2nd closest sequenced relative to KRP1	^28^

**Figure 1:**
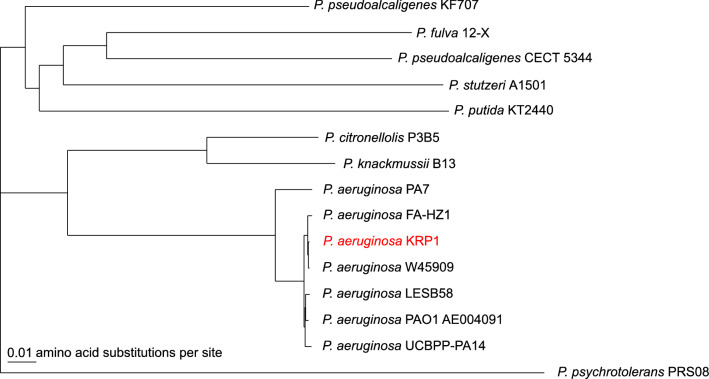
Phylogenetic tree of six fully sequenced *P. aeruginosa* strains and eight other *Pseudomonas* species. The tree was calculated using the EDGAR platform^24,25^ out of a core of 1,537 genes per genome comprised of 532,537 amino acid-residues per genome.

In the original study^20^, isolate KRP1 showed the highest similarity BLAST hit with *Pseudomonas aeruginosa* ATCC 27853 at 95% identity along a 197 bp fragment of the 16S rRNA gene. To re-evaluate its phylogenetic relationship within the *Pseudomonas* genus the average nucleotide identity (ANI) percentage of the KRP1 genome was calculated with respect to 105 fully sequenced *P. aeruginosa* strains and 8 other *Pseudomonas* species (Table S2). When compared to the *P. aeruginosa* species, all ANI values are well above the accepted species threshold of 95–96%. For the eight other closely related *Pseudomonas* species, ANI values range between 80.4% (*P.* *citronellolis* P3B5) and 74.4% (*P.* *psychrotolerans* PRS08). Besides this nucleotide based comparison, a phylogenetic tree was built based on a core of 1,537 genes per genome, each comprised of 532,537 amino acid residues (Figure S1). For better visualization, a reduced version of the tree containing only the eight non-*aeruginosa* species and six *P. aeruginosa* strains is shown (Fig. 1). The phylogenetic analysis clearly marks the strain KRP1 as a representative of the species *P. aeruginosa* and shows a clear distinction of the strain towards other members of the same genus.

### *P. aeruginosa* KRP1 relation to closely related *P. aeruginosa* strains

The phylogenetic trees in Figs. 1 and S1 are based on amino acid-sequences, and therefore present only non-synonymous nucleotide substitutions. For a more in depth investigation of KRP1, its genome was compared to the type strain PAO1, the frequently researched strain PA14, the highly virulent LESB58 strain and the two strains FA-HZ1 and W45909, to which KRP1 clusters most closely in the phylogenetic analyses (Table 1). They also show the same Multilocus sequence type (MLST) as KRP1, as they encode perfect homologues of all seven housekeeping genes used for the profiling by the MLST 2.0 software^26^ (*acsA, aroE, guaA, mutL, nuoD, ppsA* and *trpE;* Table S1). The other strains (PAO1, PA14 and LESB58) differ in at least four out of the seven genes. For FA-HZ1 and W45909, only their sample origins and genomes are known so far. FA-HZ1 is an environmental isolate from China, which was characterized for its dibenzofuran-degrading ability^27^, while W45909 is a clinical isolate from the USA^28^.

**Figure 2:**
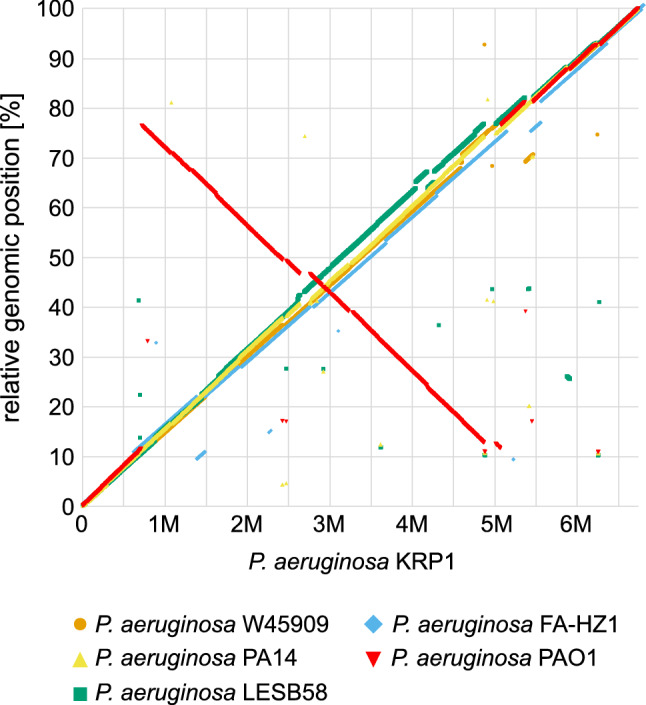
Synteny plot of the *P. aeruginosa* KRP1 strain and five other *P. aeruginosa* strains. Each dot represents a gene of KRP1 and its corresponding homologue in the respective comparative strain. x-axis shows the position within the chromosome of KRP1 and y-axis shows the relative position within the compared genome. Analysis was performed with the EDGAR platform^24,25^.

When looking at the overall genome arrangement, KRP1 shows a high degree of synteny throughout the whole genome with the strains FA-HZ1, W45909, LESB58 and PA14. Only with respect to the type strain *P. aeruginosa* PAO1 the known large-scale inversion of 70% of the genome is apparent^3,23^ (Fig. 2).

**Figure 3:**
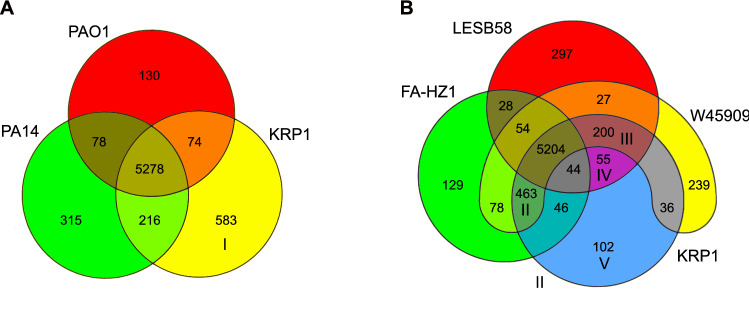
Venn diagrams showing the number of genes shared as orthologues in all possible logical combinations between different strains of *P.* *aeruginosa*. A: PAO1 [red], PA14 [green], KRP1 [yellow]; B: LESB58 [red], FA-HZ1 [green], KRP1 [blue], W45909 [yellow]. For further information regarding individual areas marked with roman numbers see text. Analysis was performed with the EDGAR platform^24,25^.

The genome of *P. aeruginosa* has a mosaic-like structure, built of a conserved core, which is interrupted by genomic islands containing variable accessory genes^7^. The numerical distribution between genes belonging to the core- and the accessory genome of the six *P. aeruginosa* strains (KRP1, PAO1, PA14, LESB58, FA-HZ1 & W45909) was analyzed using EDGAR (Fig. 3). These six strains share a common core genome of 4,978 genes, which corresponds to 76.9% (W45909)—87.3% (PAO1) of all genes annotated in the respective genomes (79% for KRP1). The core predominantly includes primary metabolism related genes, as well as genes involved in transcription and translation^29^. The core genome shared by KRP1 and the two predominantly researched strains PAO1 and PA14 consists of 5,278 genes (Fig. 3A). This is equivalent to 83.8% (KRP1)—92.6% (PAO1) of all genes annotated in the respective genomes (Table 1). There are 583 genes in KRP1, for which orthologues are not found in either of the two other strains (area I, Fig. 3A). Thus, the environmental isolate KRP1 encodes for a substantially higher number of singletons than PAO1 or PA14. As a species, *P. aeruginosa* contains another 10,000 genes, which make up the accessory genome. The overlap of genes belonging to this genome fragment in KRP1 is more pronounced with the FA-HZ1 and W45909 strains of *P. aeruginosa* (area II, Fig. 3B), which also cluster as the closest relatives of KRP1 during the phylogenetic evaluation (Figs. 1, S1). The three strains share a total of 5,667 genes, which corresponds to 89.94% of all KRP1 predicted ORFs (core + in common accessory genes). This is interesting, since all three strains originate from three different habitats and continents. This combination of core and accessory genes seems to enable the strains to thrive in a pathogenic (W45909) as well as an environmental (KRP1 and FA-HZ1) setting.

**Figure 4:**
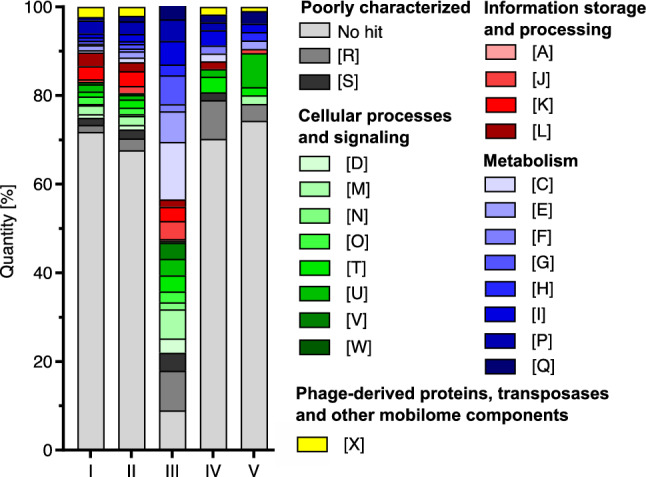
ORFs of areas I to V (groups of genes, which are singletons to KRP1 or shared by KRP1 and up to five other *P. aeruginosa* strains; see Fig. 3) classified by Clusters of Orthologous Groups (COGs) database. Category designations are as follows: [R]—General function prediction only; [S]—Function unknown; [D]**—**Cell cycle control, cell division, chromosome partitioning; [M]—Cell wall/membrane/envelope biogenesis; [N]—Cell motility; [O]—Post-translational modification, protein turnover, and chaperones; [T]—Signal transduction mechanisms; [U]—Intracellular trafficking, secretion, and vesicular transport; [V]—Defense mechanisms; [W]—Extracellular structures; [A]—RNA processing and modification; [J]—Translation, ribosomal structure and biogenesis; [K]—Transcription; [L]—Replication, recombination and repair; [C]—Energy production and conversion; [E]—Amino acid transport and metabolism; [F]—Nucleotide transport and metabolism; [G]—Carbohydrate transport and metabolism; [H]—Coenzyme transport and metabolism; [I]—Lipid transport and metabolism; [P]—Inorganic ion transport and metabolism; [Q]—Secondary metabolites biosynthesis, transport, and catabolism; [X]—Phage-derived proteins, transposases and other mobilome components.

With the highly virulent LESB58 strain, KRP1 shares a total of 5,503 genes (core + area III & IV, Fig. 3B). In an inter-species comparison of these four strains (LESB58, FA-HZ1, KRP1 & W45909; Fig. 3B), the KRP1 genome encodes the lowest number of singletons (area V, Fig. 3B). Of these 102 genes, ~ 78% did not yield a BLAST hit within the COG database, highlighting that most of the genes of this area are novel or hypothetical proteins (Fig. 4; Table S3). This high portion of unclassified genes was typical for all closer investigated overlap areas, except for the overlap of the KRP1 strain with LESB58 and W45909 (area III, Fig. 3B). Here, the majority of the genes have a metabolic function and ~ 27% are related to cellular processes and signaling, which gives a hint that the biological niches occupied by these strains seems to be similar (Fig. 4; Table S4). The strain KRP1 contains 65 singletons with respect to the other five strains. The majority of them are not classified within the COG database (Fig. 4), but are recognized as phage related proteins by the PHASTER software and are located within the identified GIs of KRP1 (Table S5).

### The accessory genome of *P. aeruginosa* KRP1

**Figure 5:**
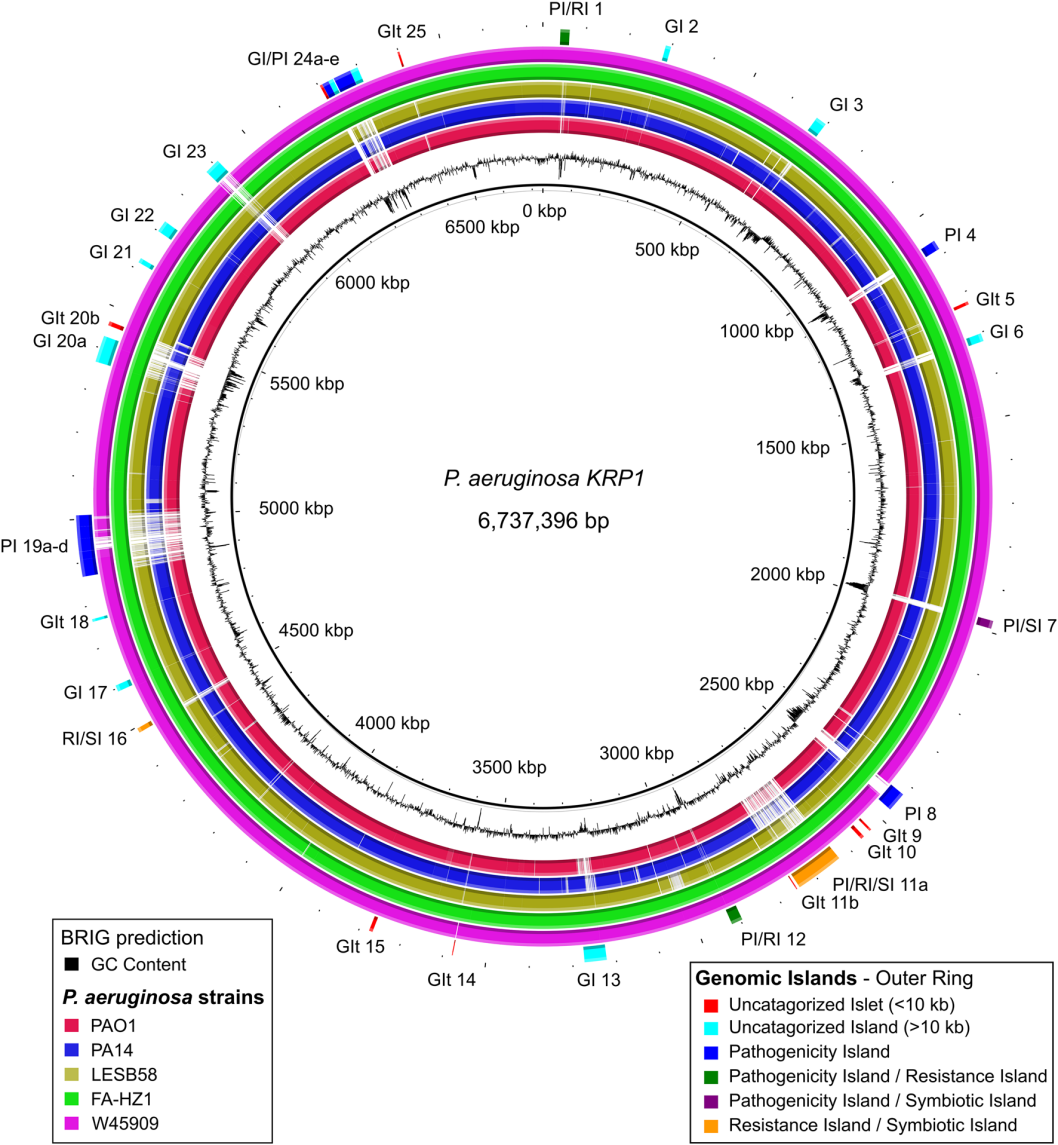
Visualization of genome plasticity in the *P. aeruginosa* KRP1 genome detected with different prediction programs. KRP1 main chromosome in comparison to selected *P. aeruginosa* genomes. Starting from the innermost circle going outwards: major- (500 kb) and minor tick (100 kb) measurements of the KRP1 genome; G + C content (black traces); BLAST comparisons of PAO1 genome against the KRP1 genome (red ring); BLAST comparisons of PA14 genome against the KRP1 genome (blue ring); BLAST comparisons of LESB58 genome against the KRP1 genome (ocher ring); BLAST comparisons of FA-HZ1 genome against the KRP1 genome (green ring); BLAST comparisons of W45909 genome against the KRP1 genome (magenta ring); combined genome plasticity prediction of SIGI-HMM^16^, IslandPath-DIMOB^17^, PHASTER^18^ and GIPSy^19^, when comparing KRP1 to PA14 as a reference (red segments: uncategorized genomic islets [GIts]; aqua segments: uncategorized genomic islands [GIs]; blue segments: pathogenicity islands [PIs]; green segments: pathogenicity/resistance islands [PI/RIs]; purple segments: pathogenicity/symbiotic islands [PI/SIs]; orange segments: resistance/symbiotic islands [RI/SIs]). Whole genome BLAST comparison and image generation was performed with BRIG^31^.

The majority of genes belonging to the accessory genome are not scattered randomly throughout the *P.* *aeruginosa* KRP1 genome, but are mainly clustered in 17 GIs and 8 GIts throughout the KRP1 genome (Table 2; Fig. 5) as detected with different bioinformatics tools (SIGI-HMM^16^, IslandPath-DIMOB^17^, PHASTER^18^ and GIPSy^19^). For some islands only different subparts were detected by the programs. If the subparts were confirmed via manual inspection to be part of the same island, they were numbered with a-e. This means that also the area in between the different sub-islands can be considered part of the accessory genome of *P. aeruginosa* KRP1. Multiple known GIs of *P. aeruginosa* were not detected by any of the used software tools but instead were determined via manual scanning of the genome. This highlights on the one hand, the usefulness of the multiple program approach for detection of putative genomic islands within a novel sequenced strain. On the other hand, it shows that the detection algorithms of the programs are not perfect and by just relying on them, relevant information might be overlooked. It is therefore crucial to complement the in silico analysis by implementing previously reported results to obtain a comprehensive view of the genomic structure of a newly sequenced strain.

**Table 2:** Summary of genomic islands predictions in *P. aeruginosa* KRP1.

Genomic island	Start position (bp)	Stop position (bp)	Size (bps)	KRP1 locus tag (number of ORFs)	RGP*	Prediction method
PI/RI 1	40,389	61,808	21,419	KRP1_00205**—**KRP1_00235 (7)	RGP46	2 and 4
GI 2	285,777	298,203	12,426	KRP1_01295—KRP1_01335 (9)	RGP2	5
GI 3	671,911	697,058	25,147	KRP1_03145**—**KRP1_03300 (32)	RGP3/4	1 and 3
PI 4	1,063,975	1,085,974	21,999	KRP1_05045**—**KRP1_05145 (21)	RGP88	1, 2, 4 and 5
GIt 5	1,222,896	1,230,252	7356	KRP1_05780—KRP1_05780 (1)	RGP89	5
GI 6	1,302,346	1,320,820	18,474	KRP1_06140**—**KRP1_06225 (17)	RGP36	1 and 2
PI/SI 7	1,973,098	1,991,464	18,366	KRP1_09255**—**KRP1_09325 (54)	RGP31	2 and 4
PI 8	2,424,758	2,470,270	45,512	KRP1_11590**—**KRP1_11760 (36)	RGP28	1, 2, 3, 4 and 5
GIt 9	2,533,287	2,538,355	5068	KRP1_12025—KRP1_12060 (8)	–	2 and 5
GIt 10	2,556,402	2,564,724	8322	KRP1_12155—KRP1_12190 (8)	RGP71	5
PI/RI/SI 11a	2,632,036	2,744,677	112,641	KRP1_12500—KRP1_13040 (109)	RGP27	1, 2, 4 and 5
GIt 11b	2,751,082	2,753,517	2435	KRP1_13080—KRP1_13095 (4)	RGP27	2
PI/RI 12	2,895,779	2,921,721	25,942	KRP1_13740—KRP1_13765 (7)	RGP25	4 and 5
GI 13	3,221,391	3,272,809	51,418	KRP1_14895—KRP1_15110 (44)	RGP23	1, 2, 4 and 5
GIt 14	3,577,280	3,579,282	2002	KRP1_16510—KRP1_16510 (1)	RGP52	5
GIt 15	3,769,299	3,777,692	8393	KRP1_17360—KRP1_17400 (9)	–	5
RI/SI 16	4,485,821	4,496,553	10,732	KRP1_20830—KRP1_20870 (9)	RGP9	4
GI 17	4,592,095	4,616,393	24,298	KRP1_21355—KRP1_21485 (27)	RGP7	1 and 5
GIt 18	4,762,338	4,768,531	6193	KRP1_2211—KRP1_22245 (8)	RGP6	5
PI 19a	4,867,542	4,906,902	39,360	KRP1_22720—KRP1_22960 (49)	RGP5	1, 3 and 4
PI 19b1	4,906,929	4,925,297	18,368	KRP1_22965—KRP1_23060 (20)	RGP5/41	1 and 4
PI 19c	4,925,522	4,955,315	29,793	KRP1_23065—KRP1_23155 (19)	RGP41	2 and 4
PI 19b2	4,955,299	4,983,156	27,857	KRP1_23160—KRP1_23310 (31)	RGP5/41	1, 2 and 4
PI 19d	4,983,197	5,009,461	26,264	KRP1_23315—KRP1_23425 (23)	RGP5	1, 2, 3 and 4
GI 20a	5,366,804	5,428,778	61,975	KRP1_25040—KRP1_25385 (70)	RGP41	1, 2 ,3, 4 and 5
GIt 20b	5,455,015	5,464,821	9807	KRP1_25540—KRP1_25565 (6)	RGP41	2, 4 and 5
GI 21	5,615,479	5,626,409	10,930	KRP1_26250—KRP1_26310 (13)	–	5
GI 22	5,700,164	5,727,413	27,249	KRP1_26660—KRP1_26790 (7)	–	5
GI 23	5,875,381	5,911,730	36,349	KRP1_27515—KRP1_27765 (51)	–	1, 3, 4, 5
GIt 24a	6,203,408	6,209,504	6096	KRP1_29015—KRP1_29040 (6)	–	5
PI 24b	6,209,865	6,225,427	15,562	KRP1_29045—KRP1_29090 (10)	RGP62	1, 2, 4 and 5
GI 24c	6,225,700	6,237,151	11,451	KRP1_29095—KRP1_29150 (12)	–	5
PI 24d	6,239,438	6,281,035	41,597	KRP1_29155—KRP1_29380 (46)	RGP87	1, 3, 4 and 5
GI 24e	6,281,429	6,299,576	18,147	KRP1_29385—KRP1_29450 (14)	–	5
GIt 25	6,397,652	6,402,302	4650	KRP1_29920—KRP1_29930 (3)	–	2

GI: genomic island (> 10 kb), GIt: genomic islets (< 10 kb), PI: pathogenicity island, RI: resistance island, SI: symbiotic islands.

Prediction method: 1: IslandPath-DIMOB^17^; 2: SIGI-HMM^16^; 3: PHASTER^18^; 4: GIPSy^19^; 5: manual blast against previously described *P. aeruginosa* GIs.

*Reported regions of genomic plasticity**—**RGPs 1–62^30^; RGPs 63–80^32^; RGPs 81–86^15^; RGPs 87–89^33^.

Since the overall average G + C content of *P. aeruginosa* KRP1 is at 66.3% (Table 1) and therefore considered G + C-rich, genes acquired through horizontal gene transfer usually have a lower G + C content (black ring in Fig. 5). After integration of the foreign DNA into the chromosome, it is subject to the same selective evolutionary pressure as the rest of the host chromosome. Thus, over time it is likely to lose the sequence compositional differences, making it undistinguishable from genomic material originating from *P. aeruginosa*^7^. These regions are therefore not detected by GI prediction software targeting differences in sequence composition. In the case of the 17 putative GIs and 8 putative GIts in KRP1, most have a notably lower G + C content compared to the surrounding core genome and are therefore of rather young evolutionary origin. Several of the homologous PAGI and LESGI GIs in KRP1, in contrast, were not detected by any of the used algorithms, which might point to an evolutionary older event of acquisition of these GIs and GIts (Tables 2, 4).

GIs and GIts tend to integrate in certain genomic loci termed “regions of genomic plasticity (RGPs)”^30^, which mark locations where integration of foreign DNA into the *P. aeruginosa* genome have been previously reported to happen with increased frequency. For the majority of GIs and GIts, a specific RGP could be assigned (Table 2). In *P. aeruginosa* KRP1 all functional classes of GIs^19^ are found, except for MIs (Table 2; Fig. 5). Since it is not necessary that each single gene of the respective GI falls into the respective category, some GIs are placed in more than one category.

The genome of KRP1 was also analyzed to identify which version of the four known replacement islands (pilin/pilin modification, flagellin glycosylation island, O-antigen gene cluster, and pyoverdine production) are encoded, as these traits represent critical determinants for the fitness and virulence of an individual *P. aeruginosa* strain^7^ (Table 3). A replacement island contains the same functional content and occupies nearly always the same genomic loci within the *P.* *aeruginosa* core genome. Intriguingly, the specific genetic sequence of each island is highly diverse between strains^34,35^. The gene loci of the O-antigen gene cluster and the flagellin glycosylation replacement island are part of the PI/SI 7 and the RI/SI 16, respectively. The pyoverdine locus is located between PI/RI 12 and GI 13, while the pilin modification genes are situated between PI 19 and GI 20. Both are not identified by the different genomic island detection programs. It is remarkable that KRP1 shares all four replacement islands subgroups with strains FA-HZ1 and W45909. In contrast, it only shares the pyoverdine subgroup with PAO1 and PA14. Variations in the pyoverdine locus have been mainly associated with different environmental fitness, as they are an entry target for pyocins, bacterially produced phage-like molecules with antibacterial properties^36^. The other three loci (pilin/pilin modification, flagellin glycosylation and O-antigen modification) have been linked to virulence properties of strains before^37–44^. The common group-I pilin variant expressed by KRP1 has been linked increasingly to cystic fibrosis environments^37^. As O-antigens, pili and flagella are recognized targets for phage entry and the host immune system, keeping different varieties of the same gene locus is thought to be a defense mechanism of *P. aeruginosa*^7^. In the case of KRP1, the intact JBD93 bacteriophage, which was detected as GI 23 (92% identity over 86% of the query length with the PHASTER software), uses O-antigen mediated infection^45^. Since PAO1 and PA14 encode the genes for different O-antigen serotypes (Table 3), they are likely no targets for JBD93. Therefore, almost all of the 51 ORFs encoded in GI 23 are unique to KRP1 in the inter-strain comparison (area I; Fig. 3A). Even though the closely related FA-HZ1 and W45909 strains also have the O1-serotype, the prophage is not encoded in their genome. Further, its integration disrupts the MdlC benzoylformate decarboxylase locus (PA14_64770), which has not been recognized as a RGP in *P. aeruginosa* before. This leads us to believe that this prophage integration into the KRP1 genome is a recent evolutionary event. Besides GI 23, the PHASTER software^18^ identifies and annotates six more prophages throughout the KRP1 genome (Table S5). All of the detected sequences can be assigned to specific GIs/GIts and were also recognized by the other genomic island detection programs tested. In general, PHASTER is not a classical GI detection software, but as the integration of a phage into a host genome is a form of horizontal gene transfer, they are part of the accessory genome of the host organism^7^. Usually other GI prediction tools will also recognize the GIs containing the putative prophage sequences, as their G + C content often differs from the one of the host, which software like GIPSY^19^ will detect. At the same time, prophages might go undetected, if by chance their G + C content is close to the nucleotide usage of the host. In these cases, PHASTER can lead to additional, otherwise undetected hits, since it utilizes a BLASTP comparison of the query genome with a frequently updated prophage sequence database^18,46^. Hence, phage related ORFs and proteins will be recognized on the basis of their sequence rather than their properties, like codon usage or G + C content by PHASTER. The software classified four out of the seven prophages of KRP1 as intact, hence their genome contains all the necessary parts to be a complete phage and therefore to also leave the genome again. It will be interesting to see what the functional role of these prophages in the lifestyle of *P. aeruginosa* KRP1 is, as they are known to be crucial for the fitness of *P. aeruginosa* under certain conditions^15,47^. These prophages might also relate to the absence of a detectable intact CRISPR-Cas defense system in the KRP1 strain^22,48^. This phenomenon has been previously recognized in other *P. aeruginosa* strains and likely relates to the increased ability of the strains to acquire antibiotic resistances through mobile elements^49^. For KRP1, the CRISPRCasFinder software detected two sets of one spacer sequence each surrounded by direct repeats. These putative spacers are not located within any of the detected GIs/GIts.

**Table 3:** Replacement islands in *P. aeruginosa.*

Replacement island	Number of subgroups	RGP*	PAO1	PA14	LESB58	FA-HZ1	W45909	KRP1
O-antigen biosynthetic locus	20^50^	RGP31	O5^51^	O10^3^	O6^52^	O1 (this study)	O1 (this study)	O1 (this study)
Pyoverdine locus	3^53^	RGP73	Type I^34^	Type I^34^	Type III^12^	Type I (this study)	Type I (this study)	Type I (this study)
Pilin and pilin modification genes	5^37^	RGP60	Group II^37^	Group III^37^	Group I^54^	Group I (this study)	Group I (this study)	Group I (this study)
Flagellin glycosylation island	2^55^	RGP9	b-type^55^	b-type^40^	b-type^56^	a-type (this study)	a-type (this study)	a-type (this study)

*RGPs 1–62^30^; RGPs 63–80^32^.

Of the GIs recognized by the prediction software packages, PI/RI 1, GI 3, PI/RI 12 and GI 17 share a large portion of their nucleotide sequence with the other investigated *P. aeruginosa* genomes (e.g., with PA14: 50%, 80%, 80% and 90%, respectively). On the other hand, unique putative genes within these islands are assigned to only one of the analyzed strains and their integration into the core genome could be traced to a specific known RGP (Table 2). This classifies them as valid regions of the accessory genome of *P. aeruginosa.*

Frequently, GI integration is observed downstream of a tRNA^57,58^. The 3′-ends of tRNAs carry *attB* sites, which are recognized and used for site-specific recombination between an integrative and conjugative element (ICE) and the main chromosome. Overall, the integration of PI 8, GI 11, RI/SI 16, GI 17, PI 19, GI 20 and PI 24b&d occurred just downstream of specific tRNAs within the KRP1 genome. Of these islands, GI 11, PI 19 and GI 20 belong to the same family of *P. aeruginosa* GIs, which are marked by their bipartite structure. While the first segment, downstream of the tRNA, contains strain-specific cargo ORFs, the second part shows a high degree of sequence similarity between the strains^15,57^ and mainly encodes structural and mobility-related genes, as well as genes for conjugal transfer^9^. Cargo genes of GI 11 include heavy metal resistance genes, genes for metabolic enzymes and enzymes used for the formation and altering of nucleic acids, transcription regulators, a two-component system, as well as an antibiotic resistance gene. While the here analyzed cargo genes are KRP1-specific with respect to detected and analyzed GIs (i.e., PAGI-2, PAGI-3 and LESGI-3^15,57^), they share 99% sequence identity with 13 of the 105 *P. aeruginosa* isolates used for phylogenetic comparison (Table S2). Hence, the entire genomic island is part of the genomic make-up of multiple previously sequenced *P. aeruginosa* cultures. These include the previously mentioned FA-HZ1 and W45909 strains. We hypothesize that this set of cargo genes form a unit, which contributes to the successful survival of *P. aeruginosa* in certain habitats.

### Genomic resemblance of KRP1 to highly virulent *P. aeruginosa* strains

The production of many known important virulence factors of *P. aeruginosa* is encoded within the core genome^59^. While no apathogenic variants of the species have been reported so far, a strong intraspecies gradient of virulence is observed, ranging from highly infective to only mellow virulent strains^4,13^. This phenomenon is likely linked to the varying accessory genome of the variants. Based on the genome analysis presented here, overall predictions of the virulence of KRP1 are possible, which can be used as a guidance for further experiments involving this organism. *P. aeruginosa* KRP1 contains an array of genomic elements that are found in the highly virulent strains PSE9^13,33^ and LESB58^12,15,60^ (Table 4). Unfortunately, no complete genome sequence is available for PES9 yet, so it could not be included in the full genome comparison. However, some of the shared GIs have been shown to be the source of the strain dependent virulence within the *P. aeruginosa* species^13–15^. KRP1 encodes all seven genomic islands found in the clinical isolate PSE9^13,33^ (Table 4). The PSE9 strain originated from a patient with ventilator-associated pneumonia isolated at a hospital in Barcelona, Spain in the mid-1990s^61^. It was found to be the most virulent out of 35 strains in a mouse model of acute pneumonia^62^. So far, two studies were able to link the increased virulence of PSE9 directly to PAGI-5 and PAGI-9^13,14^. Since KRP1 contains both of the mentioned islands, an increased virulence similar to the levels of PSE9 can be anticipated. PAGI-9 of PSE9 and GIt 5 of KRP1, respectively, consist of 6581 bps and one large ORF, which was identified as a *Rhs* (rearrangement hot spot) element^33^. Similarly, PAGI-10 is a *Rhs* element of PSE9, which is also found within KRP1 (PI/RI 9). The nucleotide sequence of these proteins generally has a bipartite structure composed of a long G + C rich core and a relatively G + C poor tip sequence. While the core sequence is intra- and interspecies highly conserved, the tip is rather variable. The fact that the strains PSE9 and KRP1 show sequence identity over the entire length of the ORFs and not only in the conserved core shows the close genomic relationship between the hyper virulent PSE9 and KRP1.

**Table 4:** Genomic Islands (GIs) and genomic islets (GIts) in different *P. aeruginosa* strains.

	Location within KRP1	Sequence identity (query length)
**PSE 9 GIs**		
PAGI-5	GI 20	99.98% (98%)
PAGI-6	PI 24d	99.98% (100%)
PAGI-7	PI 4	100% (100%)
PAGI-8	PI 24b	99.99% (100%)
PAGI-9	GIt 5	100% (100%)
PAGI-10	PI/RI 9	99.97% (100%)
PAGI-11	GIt 14	100% (100%)
**LESB58 GIs**		
LESGI-1	PI 8	98.62% (96%)
LESGI-3	GI 11	99.54% (65%)
LESGI-4	GI 13	99.61% (98%)
LESGI-6	GI 2	99.36% (100%)
LESGI-8	GIt 9	99.41% (100%)
LESGI-9	GIt 10	99.75% (100%)
LESGI-12	GIt 15	99.55% (100%)
LESGI-13	GI 17	99.60% (100%)
LESGI-14	GIt 18	99.56% (100%)
LESGI-15	GI 21	99.73% (100%)
LESGI-16	GI 22	99.62% (100%)
LESGI-17	GI 24	99.59% (96%)
LES-prophage 4	GI 23	89.31% (73%)

GIs of strain PSE9 and selected GIs of strain LESB58 and their corresponding GI and GIts, as well as sequence similarity within the strain KRP1.

PAGI-11 of PSE9 (GIt 14 in KRP1) is only 2003 bps long and located at RGP 52 (Table 4) and while Battle et al.^33^ did not find any ORFs contained, the Prokka pipeline^63^, applied to the KRP1 genome, predicts the hypothetical protein KRP1_16515. The G + C content of just 43.19% is far below the average of the KRP1 genome (i.e. 66.3%). Other strains are known to contain larger GIs encoding mobile element related genes at this specific genomic locus^30^. Therefore, PAGI-11 might have been a larger genomic island in the past, which was partially lost over time in PSE9 and KRP1.

Further, PSE9 and KRP1 share the same O-antigen type O1 (Table 3). The O-antigen type of the outer membrane lipopolysaccharide (LPS) layer has been previously linked to the virulence of *P. aeruginosa*, but most studies consider the serotype of the type strain PAO1 (type O5)^43^. Both strains are also *exoS* positive and *exoU* negative, a genotype that has been linked to an invasive phenotype^64^. Since no full genome sequence of PSE9 is available so far, a deeper in silico comparison between both strains is currently impossible.

Besides PSE9, the *P. aeruginosa* strain KRP1 shows substantial similarities in its accessory genome with the LESB58 strain, an aggressive pathogen of a cystic fibrosis patient from Liverpool in 1988^12,15,60^ (Table 4). The strain is beta-lactam-resistant^60^, exhibits enhanced survival on dry surfaces^65^, shows an increased patient morbidity^66^, and overexpression of parts of the quorum sensing regulon during early growth phases (e.g., LasA, elastase, and pyocyanin)^67,68^. It is also known to replace previously established *P. aeruginosa* strains due to its aggressive nature, thereby causing a superinfection^69^. A LES isolate has even been reported to have infected the non-CF parents of a CF patient^70^. While the complete reasons for its increased virulence are still partially unknown, a lot of the responsible factors are thought to be driven by the accessory genome of the strain^12,15^. These LESGI termed genomic islands differentiate the LES strain from other *P. aeruginosa* strains. Of the 17 known LESGIs and six LESGI-prophages, the genome of KRP1 contains 12 LESGIs and one prophage (Table 4). The majority of the shared GIs and GIts were found via manual search rather than by the applied software programs (Table 2). LESGI-6 to LESGI-17 were first detected by Jani et al.^12^. The authors used a genome segmentation approach to identify genomic regions of foreign origin within the LESB58 strain. This technique varies from the ones used in this study and therefore different putative GIs and GIts were detected. The authors could show that these GI encode for additional virulence factors (LESGI-6, -8, -13, and -15) as well as drug and metal resistance cassettes (LESGI-12 and -17). LESGI-9, -16, and -17 add additional versatility to the LESB58 metabolic repertoire^12^. Since KRP1 encodes all of these GIs as well, it is very likely that it employs their functions and therefore shows an increased virulence potential, similar to the LESB58 strain.

In contrast, the two strains showing the closest ANI identity and phylogenetic relationship with KRP1 are *P. aeruginosa* strain FA-HZ1 and W45909 (Fig. 1). FA-HZ1 is a dibenzofuran-degrading isolate from China^27^ while W45909 is a clinical isolate from the USA^28^. All but three identified GIs in KRP1 are also present in these two most related strains (PI 8, PI 19 and GI 23 for W45909 and GI 23 for FA-HZ1). This provides circumstantial evidence that the genomic repertoire of *P. aeruginosa* KRP1 is likely to sustain a pathogenic as well as an apathogenic lifestyle in nature. While their genetic information is available, no further studies have been performed with either of these strains but we stand to believe that they will also show an increased virulence like PSE9, LESB58 and likely KRP1.

## Conclusion

The genome of the BES isolate *Pseudomonas* sp. KRP1 was de novo sequenced and analyzed in depth for its phylogenetic relationship within the *Pseudomonas* clade. Due to the sequence composition of its core genome, it could clearly be assigned to belong to the *P.* *aeruginosa* species. Its closest relatives are two recently sequenced strains from China (FA-HZ1)^27^ and the USA (W45909)^28^.

The accessory genome of KRP1 was thoroughly analyzed. Using four different prediction programs, 17 putative genomic islands and 8 putative genomic islets were detected. This analysis was extended by mining for the 44 GI complexes previously described in *P. aeruginosa*^9–12^. Most of the GIs and GIts could clearly be assigned to a known RGP (Table 2). The majority of the KRP1 singletons, with respect to the strains PAO1, PA14, FA-HZ-1, W45909 and LESB58, are contained in these islands, marking them as the main source of genome divergence between the strains.

Utilizing the increased amount of sequencing data made publicly available in the past decade, it is possible to make in silico based educated prediction towards the virulence potential of a newly isolated strain of *P.* *aeruginosa*. Hence, it decreases the need for laborious trial and error type wet lab experiments and animal testing. The hurdle to get permission to do animal experiments, for example in Germany, is fairly high and not every lab facility has the necessary infrastructure for this type of investigations. With an in silico investigation, like the one presented in the manuscript, also these labs have the option to easily obtain valuable information on the strain they investigate. This kind of educated knowledge about the expected pathogenicity of an isolate can as well help in the daily handling of the organism in the labs itself. As every *P. aeruginosa* has a certain pathogenic potential, they are all classified as risk group two and should all be handled with the same caution in the lab. But the degree of virulence actually varies substantially between strains^4^. For species isolated from e.g., infection scenarios, a high virulence is obvious. Instead, KRP1 is an environmental isolate that was spotted because it dominated in a natural mixed culture biofilm^20^. By being aware of the potentially high virulence of the organisms, personal safety measurements can be increased to avoid an accidental exposition of the organism. Using publicly available data and their integration with own research data can help to substantially speed up research in the future and to draw wider, more general conclusions. The true degree to which the individual GIs and GIts contribute to virulence of the strain is still to be determined and proves to be a rather difficult task since virulence in *P. aeruginosa* is known to be combinatorial^3,71^.

## Methods

### Strain and medium

*P. aeruginosa* KRP1 was isolated from a microbial fuel cell setup at the Laboratory of Microbial Ecology and Technology (LabMET) at Ghent University (deposited into the Belgian Co-ordinated Collections of Microorganisms, BCCM; strain number LMG 23,160)^20^. Cultures were grown in shake flasks in Luria Broth medium at 37 °C, 200 rpm shaking.

### DNA sequencing

Genomic DNA of *P. aeruginosa* KRP1 was isolated via phenol–chloroform extraction^72^^; mod.^. Besides a purity check on a NanoDrop One/OneC Microvolume-UV–Vis-Spectrophotometer (Thermo Fisher Scientific) and an integrity check via agarose gel electrophoresis, the concentration of isolated genomic DNA was estimated via a PicoGreen dsDNA quantification assay (Quant-iT PicoGreen dsDNA Assay Kit, Thermo Fisher Scientific). The measurement for this assay was done with a Synergy Mx microplate reader (BioTek) in 96-well plates using an excitation wavelength of 480 nm, an emission wavelength of 520 nm, a scan width of 9.0 and an overflow value of 80.

For shotgun library preparation, 1 µg of chromosomal DNA was used (TruSeq DNA PCR-Free Library Preparation Kit, Illumina). Samples were sequenced on an Illumina MiSeq system using the MiSeq Reagent Kit v3 for 600 cycles. The data (542.3 Mb equaling ~ 81.3 × coverage) were assembled using Newbler v.2.8 (Roche), which resulted in 58 scaffolds containing 94 contigs. Gap closure was conducted with a MinION Mk1B Sequencer from Oxford Nanopore Technologies. For this second shotgun library, 2 μg of genomic DNA was used as starting material. Size-selected DNA-fragments of 5 to 50 kb were used to create a 1D^2^ sequencing library according to the manufacturer’s instructions (1D^2^ Sequencing Kit (R9.5), Oxford Nanopore Technologies). The sequencing library was run on a R9.5 flowcell for 3 h. Base calling and data conversion to FastQ was performed using Albacore v1.2.4^73^. The resulting 72.4 Mb (12 × coverage) sequencing data were assembled with Canu v1.5^74^. After assembly, the resulting 23 contigs were polished with the short Illumina reads using PILON^75^. The final assembly was done manually using Consed^76^ to combine the contigs of the Newbler and Canu assemblies, as well as to resolve any discrepancies between the two different assemblies. This hybrid approach of a short read- (Newbler) and long read assembler (Canu) followed by manual curation, was done to fulfill the 3C criteria of genome assembly (contiguity, correctness and completeness)^22^ to a sufficient degree. Gene prediction and annotation of the finished genome were performed using the Prokka pipeline^63^. Visualization and inspection of the annotated sequence was done in Artemis^77^.

To clarify the existence of a potential mega plasmid, a PCR using EconoTaq PLUS GREEN DNA polymerase (Lucigen) was performed. The PCR fragments were sequenced by Eurofins Genomics (Germany).

### Comparative genome analysis

For the analysis of the assembled KRP1 genome in respect to other *Pseudomonas* genomes and to find orthologous genes in related genomes, the EDGAR ("Efficient Database framework for comparative genome analyses using BLAST score Ratios")^24,25^ platform was used. Via the platform, the synteny analysis, the distribution of gene sets into core genome, accessory genome and singletons, the ANI calculations, and the phylogenetic tree generation were performed. For the phylogenetic trees, EDGAR utilizes an alignment of all core genes of every genome included in the comparison via MUSCLE^78^. This compiled alignment is the input for the neighbor-joining algorithm used by the PHYLIP package (https://evolution.genetics.washington.edu/phylip.html) to construct the phylogenetic tree. Hence, rather than being based on the 16S RNA sequence or the MLST sequences, the here presented trees are based on the entire core genome of the analyzed strains.

For functional gene classification, ORFs were checked against the Clusters of Orthologous Groups (COG) database^79^. Parameters were set to an e-value of < 1e^−10^ and 80% identity. MLST profiling was done using MLST 2.0 v2.0.4^26^. The genome of KRP1 was compared to 105 fully sequenced *P. aeruginosa* strains and 8 other *Pseudomonas* species. These represent all publicly available fully finished and closed *P. aeruginosa* genomes available from the NCBI website at the time of conducting this study. More in depth analyses were performed with the type strain PAO1 (AE004091; NC_002516.2;^23^), the frequently researched strain PA14 (UCBPP-PA14; NC_008463.1^3^), the highly virulent strain LESB58 (NC_011770.1^15^) and the two phylogenetically closest strains FA-HZ1 (NZ_CP017353.1^27^) and W45909 (NZ_CP008871.2^28^). The accession numbers of the other ~ 100 *Pseudomonas* strains used for the ANI and phylogenetic comparison can be found in Table S2.

Multiple genomic island prediction software packages were applied to analyze the KRP1 genome with respect to its genome plasticity. For GI and GIt detection the following programs were used: IslandViewer^80,81^, which incorporates the SIGI-HMM^16^ and the IslandPath-DIMOB^17^ software, and GIPSy (Genomic island prediction software)^19^. PHASTER (PHAge Search Tool—Enhanced Release) was used for identification and annotation of prophage sequences within the KRP1 genome^18,46^. Spine and AGEnt were applied for prediction of the accessory genome in its entirety^82^. Results were imaged with BRIG (BLAST Ring Image Generator)^31^. This automated GI detection was complemented by manual curration of the precise starting and stopping position of each detected island via different blast comparisons. Additionally, the genome was manually mined for any of the 44 GI complexes previously described in *P. aeruginosa*^9–12^. To evaluate the relationship of the GI content with a potential CRISPR-Cas systems in the strain, CRISPRCasFinder v1.1.2^83^ was used.

The ACT (Artemis Comparison Tool)^84^ was used for manual detection of regions of genomic plasticity (RGPs). It was also the visualization method of choice for partial and whole genome comparisons of KRP1 with different *P. aeruginosa* strains.

### Accession code

The dataset (full genome data of *P. aeruginosa KRP1*) generated and analysed during the current study is available in the NCBI BioProject database repository, (http://www.ncbi.nlm.nih.gov/bioproject/) under accession number CP046069. It is part of the ElectricMicrobe100 Umbrella BioProject, which can be accessed via PRJNA417841.

## Supplementary information

Supplementary Information 1.

## References

[1] Silby MW, Winstanley C, Godfrey SA, Levy SB, Jackson RW (2011). *Pseudomonas* genomes: diverse and adaptable. FEMS Microbiol. Rev..

[2] de Bentzmann S, Plesiat P (2011). The *Pseudomonas aeruginosa* opportunistic pathogen and human infections. Environ. Microbiol..

[3] Lee DG (2006). Genomic analysis reveals that *Pseudomonas aeruginosa* virulence is combinatorial. Genome Biol..

[4] Hilker R (2015). Interclonal gradient of virulence in the *Pseudomonas aeruginosa* pangenome from disease and environment. Environ. Microbiol..

[5] He J (2004). The broad host range pathogen *Pseudomonas aeruginosa* strain PA14 carries two pathogenicity islands harboring plant and animal virulence genes. Proc. Natl. Acad. Sci. USA.

[6] Bennett PM (2004). Genome plasticity: insertion sequence elements, transposons and integrons, and DNA rearrangement. Methods Mol. Biol..

[7] Kung VL, Ozer EA, Hauser AR (2010). The Accessory genome of *Pseudomonas aeruginosa*. Microbiol. Mol. Biol. Rev..

[8] Tümmler, B. In *Pseudomonas: Volume 4 Molecular Biology of Emerging Issues* (eds J.-L. Ramos & R. C. Levesque) 35–68 (Springer US, 2006).

[9] Klockgether J, Cramer N, Wiehlmann L, Davenport CF, Tummler B (2011). *Pseudomonas aeruginosa* genomic structure and diversity. Front. Microbiol..

[10] Silveira MC, Albano RM, Asensi MD, Carvalho-Assef APDA (2016). Description of genomic islands associated to the multidrug-resistant *Pseudomonas aeruginosa* clone ST277. Infect. Genet. Evol..

[11] Hong JS, Yoon EJ, Lee H, Jeong SH, Lee K (2016). Clonal dissemination of *Pseudomonas aeruginosa* sequence type 235 isolates carrying blaIMP-6 and emergence of blaGES-24 and blaIMP-10 on novel genomic islands PAGI-15 and -16 in South Korea. Antimicrob. Agents Chemother..

[12] Jani M, Mathee K, Azad RK (2016). Identification of novel genomic islands in liverpool epidemic strain of *Pseudomonas aeruginosa* using segmentation and clustering. Front. Microbiol..

[13] Battle SE, Meyer F, Rello J, Kung VL, Hauser AR (2008). Hybrid pathogenicity island PAGI-5 contributes to the highly virulent phenotype of a *Pseudomonas aeruginosa* isolate in mammals. J. Bacteriol..

[14] Kung VL (2012). An *rhs* gene of *Pseudomonas aeruginosa* encodes a virulence protein that activates the inflammasome. Proc. Natl. Acad. Sci. USA.

[15] Winstanley C (2009). Newly introduced genomic prophage islands are critical determinants of in vivo competitiveness in the Liverpool epidemic strain of *Pseudomonas aeruginosa*. Genome Res..

[16] Waack S (2006). Score-based prediction of genomic islands in prokaryotic genomes using hidden Markov models. BMC Bioinform..

[17] Hsiao W, Wan I, Jones SJ, Brinkman FSL (2003). IslandPath: aiding detection of genomic islands in prokaryotes. Bioinformatics.

[18] Arndt D (2016). PHASTER: a better, faster version of the PHAST phage search tool. Nucleic Acids Res..

[19] Soares SC (2016). GIPSy: Genomic island prediction software. J. Biotechnol..

[20] Rabaey K, Boon N, Siciliano SD, Verhaege M, Verstraete W (2004). Biofuel cells select for microbial consortia that self-mediate electron transfer. Appl. Environ. Microbiol..

[21] Bosire EM, Blank LM, Rosenbaum MA (2016). Strain- and substrate-dependent redox mediator and electricity production by *Pseudomonas aeruginosa*. Appl. Environ. Microbiol..

[22] Molina-Mora JA, Campos-Sánchez R, Rodríguez C, Shi L, García F (2020). High quality 3C de novo assembly and annotation of a multidrug resistant ST-111 *Pseudomonas aeruginosa* genome: benchmark of hybrid and non-hybrid assemblers. Sci. Rep..

[23] Stover CK (2000). Complete genome sequence of *Pseudomonas aeruginosa* PAO1, an opportunistic pathogen. Nature.

[24] Blom J (2009). EDGAR: A software framework for the comparative analysis of prokaryotic genomes. BMC Bioinform..

[25] Blom, J. *et al.* EDGAR 2.0: an enhanced software platform for comparative gene content analyses. *Nucleic Acids Res.* **44**, W22-W28, (2016). 10.1093/nar/gkw255 PMC4987874 27098043

[26] Larsen MV (2012). Multilocus sequence typing of total-genome-sequenced bacteria. J. Clin. Microbiol..

[27] Ali F, Hu H, Xu P, Tang H (2017). Complete genome sequence of *Pseudomonas aeruginosa* FA-HZ1, an efficient dibenzofuran-degrading bacterium. Genome Announc..

[28] Yan J (2017). Bow-tie signaling in c-di-GMP: Machine learning in a simple biochemical network. PLoS Comput. Biol..

[29] Valot B (2015). What it takes to be a *Pseudomonas aeruginosa*? The core genome of the opportunistic pathogen updated. PLoS ONE.

[30] Mathee K (2008). Dynamics of *Pseudomonas aeruginosa* genome evolution. Proc. Natl. Acad. Sci. USA.

[31] Alikhan N-F, Petty NK, Ben Zakour NL, Beatson SA (2011). BLAST ring image generator (BRIG): simple prokaryote genome comparisons. BMC Genomics.

[32] Roy PH (2010). Complete genome sequence of the multiresistant taxonomic outlier *Pseudomonas aeruginosa* PA7. PLoS ONE.

[33] Battle SE, Rello J, Hauser AR (2009). Genomic islands of *Pseudomonas aeruginosa*. FEMS Microbiol. Lett..

[34] Smith EE, Sims EH, Spencer DH, Kaul R, Olson MV (2005). Evidence for diversifying selection at the pyoverdine locus of *Pseudomonas aeruginosa*. J. Bacteriol..

[35] Subedi D, Kohli GS, Vijay AK, Willcox M, Rice SA (2019). Accessory genome of the multi-drug resistant ocular isolate of *Pseudomonas aeruginosa* PA34. PLoS ONE.

[36] Baysse C (1999). Uptake of pyocin S3 occurs through the outer membrane ferripyoverdine type II receptor of *Pseudomonas aeruginosa*. J. Bacteriol..

[37] Kus JV, Tullis E, Cvitkovitch DG, Burrows LL (2004). Significant differences in type IV pilin allele distribution among *Pseudomonas aeruginosa* isolates from cystic fibrosis (CF) versus non-CF patients. Microbiology.

[38] Arora SK, Neely AN, Blair B, Lory S, Ramphal R (2005). Role of motility and flagellin glycosylation in the pathogenesis of *Pseudomonas aeruginosa* burn wound infections. Infect. Immunol..

[39] Kuang Z (2011). The *Pseudomonas aeruginosa* flagellum confers resistance to pulmonary surfactant protein-A by impacting the production of exoproteases through quorum-sensing. Mol. Microbiol..

[40] Verma A (2006). Glycosylation of b-type flagellin of *Pseudomonas aeruginosa:* structural and genetic basis. J. Bacteriol..

[41] Meyer JM, Neely A, Stintzi A, Georges C, Holder IA (1996). Pyoverdin is essential for virulence of *Pseudomonas aeruginosa*. Infect. Immunol..

[42] Cornelis, P. & Dingemans, J. *Pseudomonas aeruginosa* adapts its iron uptake strategies in function of the type of infections. *Front. Cell Infect. Microbiol.* **3**, (2013). 10.3389/fcimb.2013.00075 PMC3827675 24294593

[43] Pier GB (2007). *Pseudomonas aeruginosa* lipopolysaccharide: a major virulence factor, initiator of inflammation and target for effective immunity. Int. J. Med. Microbiol.

[44] Augustin DK (2007). Presence or absence of lipopolysaccharide O antigens affects type III secretion by *Pseudomonas aeruginosa*. J. Bacteriol..

[45] Bondy-Denomy J (2016). Prophages mediate defense against phage infection through diverse mechanisms. ISME J..

[46] Zhou Y, Liang Y, Lynch KH, Dennis JJ, Wishart DS (2011). PHAST: a fast phage search tool. Nucleic Acids Res..

[47] Molina-Mora JA (2020). Transcriptomic determinants of the response of ST-111 *Pseudomonas aeruginosa* AG1 to ciprofloxacin identified by a top-down systems biology approach. Sci. Rep..

[48] van der Zee A (2018). Spread of carbapenem resistance by transposition and conjugation among *Pseudomonas aeruginosa*. Front. Microbiol..

[49] Pawluk, A., Bondy-Denomy, J., Cheung, V. H., Maxwell, K. L. & Davidson, A. R. A new group of phage anti-CRISPR genes inhibits the type I-E CRISPR-Cas system of *Pseudomonas aeruginosa*. *mBio* **5**, e00896, (2014). 10.1128/mBio.00896-14 PMC3993853 24736222

[50] Liu PV, Wang S (1990). Three new major somatic antigens of *Pseudomonas aeruginosa*. J. Clin. Microbiol..

[51] Burrows LL, Charter DF, Lam JS (1996). Molecular characterization of the *Pseudomonas aeruginosa* serotype O5 (PAO1) B-band lipopolysaccharide gene cluster. Mol. Microbiol..

[52] Lam JS, Taylor VL, Islam ST, Hao Y, Kocíncová D (2011). Genetic and functional diversity of *Pseudomonas aeruginosa* Lipopolysaccharide. Front. Microbiol..

[53] Meyer JM (1997). Use of siderophores to type pseudomonads: the three *Pseudomonas aeruginosa* pyoverdine systems. Microbiology.

[54] CL Giltner N Rana MN Lunardo AQ Hussain LL Burrows 2011 Evolutionary and functional diversity of the Pseudomonas type IVa pilin island Environ. Microbiol. 13 250 264 10.1111/j.1462-2920.2010.02327.x 20738375

[55] Arora SK, Bangera M, Lory S, Ramphal R (2001). A genomic island in *Pseudomonas aeruginosa* carries the determinants of flagellin glycosylation. Proc. Natl. Acad. Sci. USA.

[56] Varga JJ (2015). Genotypic and phenotypic analyses of a *Pseudomonas aeruginosa* chronic bronchiectasis isolate reveal differences from cystic fibrosis and laboratory strains. BMC Genomics.

[57] Larbig KD (2002). Gene Islands Integrated into tRNA(Gly) Genes confer genome diversity on a *Pseudomonas aeruginosa* clone. J. Bacteriol..

[58] Kiewitz C, Larbig K, Klockgether J, Weinel C, Tümmler B (2000). Monitoring genome evolution *ex vivo*: reversible chromosomal integration of a 106 kb plasmid at two tRNALys gene loci in sequential *Pseudomonas aeruginosa* airway isolates. Microbiology.

[59] Wolfgang MC (2003). Conservation of genome content and virulence determinants among clinical and environmental isolates of *Pseudomonas aeruginosa*. Proc. Natl. Acad. Sci. USA.

[60] Cheng K (1996). Spread of beta-lactam-resistant *Pseudomonas aeruginosa* in a cystic fibrosis clinic. Lancet.

[61] Hauser AR (2002). Type III protein secretion is associated with poor clinical outcomes in patients with ventilator-associated pneumonia caused by *Pseudomonas aeruginosa*. Crit. Care Med..

[62] Schulert GS (2003). Secretion of the toxin ExoU is a marker for highly virulent *Pseudomonas aeruginosa* Isolates obtained from patients with hospital-acquired pneumonia. J. Infect. Dis..

[63] Seemann T (2014). Prokka: rapid prokaryotic genome annotation. Bioinformatics.

[64] Juan C, Peña C, Oliver A (2017). Host and Pathogen Biomarkers for Severe *Pseudomonas aeruginosa* Infections. J. Infect. Dis..

[65] Panagea S, Winstanley C, Walshaw MJ, Ledson MJ, Hart CA (2005). Environmental contamination with an epidemic strain of *Pseudomonas aeruginosa* in a Liverpool cystic fibrosis centre, and study of its survival on dry surfaces. J. Hosp. Infect..

[66] Al-Aloul M (2004). Increased morbidity associated with chronic infection by an epidemic *Pseudomonas aeruginosa* strain in CF patients. Thorax.

[67] Salunkhe P (2005). A cystic fibrosis epidemic strain of *Pseudomonas aeruginosa* displays enhanced virulence and antimicrobial resistance. J. Bacteriol..

[68] Fothergill JL (2007). Widespread pyocyanin over-production among isolates of a cystic fibrosis epidemic strain. BMC Microbiol..

[69] McCallum, S. J. *et al.* Superinfection with a transmissible strain of *Pseudomonas aeruginosa* in adults with cystic fibrosis chronically colonised by *P. aeruginosa*. *Lancet* **358**, 558–560, (2001). 10.1016/s0140-6736(01)05715-4 11520530

[70] McCallum S (2002). Spread of an epidemic *Pseudomonas aeruginosa* strain from a patient with cystic fibrosis (CF) to non-CF relatives. Thorax.

[71] Harrison EM (2010). Pathogenicity islands PAPI-1 and PAPI-2 contribute Individually And Synergistically To The Virulence Of *Pseudomonas aeruginosa* strain PA14. Infect. Immunol..

[72] Altenbuchner J, Cullum J (1984). DNA amplification and an unstable arginine gene in *Streptomyces lividans* 66. Mol. Gen. Genet..

[73] Albacore v1.2.4 tool. *https: //github.com/Albacore/albacore*.

[74] Koren S (2017). Canu: scalable and accurate long-read assembly via adaptive k-mer weighting and repeat separation. Genome Res..

[75] Walker BJ (2014). Pilon: An integrated tool for comprehensive microbial variant detection and genome assembly improvement. PLoS ONE.

[76] Gordon D, Green P (2013). Consed: a graphical editor for next-generation sequencing. Bioinformatics.

[77] Rutherford K (2000). Artemis: sequence visualization and annotation. Bioinformatics (Oxford, England).

[78] Edgar RC (2004). MUSCLE: multiple sequence alignment with high accuracy and high throughput. Nucleic Acids Res..

[79] Tatusov RL, Galperin MY, Natale DA, Koonin EV (2000). The COG database: a tool for genome-scale analysis of protein functions and evolution. Nucleic Acids Res..

[80] Langille MGI, Brinkman FSL (2009). IslandViewer: an integrated interface for computational identification and visualization of genomic islands. Bioinformatics.

[81] Bertelli C (2017). IslandViewer 4: expanded prediction of genomic islands for larger-scale datasets. Nucleic Acids Res..

[82] Ozer EA, Allen JP, Hauser AR (2014). Characterization of the core and accessory genomes of *Pseudomonas aeruginosa* using bioinformatic tools Spine and AGEnt. BMC Genomics.

[83] Couvin D (2018). CRISPRCasFinder, an update of CRISRFinder, includes a portable version, enhanced performance and integrates search for Cas proteins. Nucleic Acids Res..

[84] Carver TJ (2005). ACT: the Artemis Comparison Tool. Bioinformatics.

