# Supplementary material for: Estimation of pathogenic potential of an environmental Pseudomonas aeruginosa isolate using comparative genomics
Source: Sci Rep. 2021 Jan 14;11:1370. doi: 10.1038/s41598-020-80592-8 (PMC7809047; doi:10.1038/s41598-020-80592-8)
Supplement: Supplementary file 1 — Supplementary Information 1. [file 41598_2020_80592_MOESM1_ESM.pdf]

Supplementary information for:

Estimation of pathogenic potential of an environmental *Pseudomonas aeruginosa* isolate using comparative genomics

Carola Berger[1], Christian Rückert[2], Jochen Blom[3], Korneel Rabaey[4], Jörn Kalinowski[2] and Miriam A. Rosenbaum[1,5,*]

[1] Bio Pilot Plant, Leibniz Institute for Natural Product Research and Infection Biology – Hans-Knöll-Institute (HKI), Jena, Germany

[2] Center for Biotechnology - CeBiTec, University of Bielefeld, Bielefeld, Germany

[3] Bioinformatics and Systems Biology, Justus-Liebig University Gießen, Giessen, Germany

[4] Laboratory of Microbial Ecology and Technology (LabMET), Ghent University, Ghent, Belgium

[5] Faculty of Biological Sciences, Friedrich Schiller University, Jena, Germany

* Corresponding author:
Miriam A. Rosenbaum
Beutenbergstr. 11a,
07745 Jena
Phone: +49-3641-532-1120
miriam.rosenbaum@leibniz-hki.de

Running titel: Comparative genomics for *P. aeruginosa*
Keywords: comparative genomics, genomic island, pathogenic potential, *P. aeruginosa*

<u>This supplemental material document contains:</u>

Page 2: Table S1: Multilocus sequence type (MLST) profile of *P. aeruginosa* KRP1 and 5 other selected *P. aeruginosa* strains.

Page 3: Table S2: Calculated ANI values for 104 *P. aeruginosa* strains and 8 other *Pseudomonas* strains vs. *P. aeruginosa* KRP1.

Page 4: Table S3: Clusters of Orthologous Groups classification of singleton hits of *P. aeruginosa* KRP1.

Page 5: Table S4: Absolute number of ORFs belonging to different COG database categories of area I to V (Figure 4).

Page 6: Table S5: PHASTER prediction for putative GIs of KRP1 characterized as prophages and phage-like elements.

Page 7: Figure S1: Phylogenetic tree of 105 fully sequenced *P. aeruginosa* strains.

Table S1: Multilocus sequence type (MLST) profile of *P. aeruginosa* KRP1 and 5 other selected *P. aeruginosa* strains. The profiling was done with MLST 2.0[1]. Exact sequence homologues between the genes of the strains and the respective KRP1 gene are highlighted in bold.

**Housekeeping gene**	**KRP1 Allele**	**PA14 Allele**	**PAO1 Allele**	**LESB58 Allele**	**FA-HZ1 Allele**	**W45909 Allele**
acsA	*acsA*_6	*acsA*_4	*acsA*_7	***acsA*_6**	***acsA*_6**	***acsA*_6**
aroE	*aroE*_5	*aroE*_4	***aroE*_5**	***aroE*_5**	***aroE*_5**	***aroE*_5**
guaA	*guaA*_6	*guaA*_16	*guaA*_12	*guaA*_11	***guaA*_6**	***guaA*_6**
mutL	*mutL*_7	*mutL*_12	*mutL*_3	*mutL*_3	***mutL*_7**	***mutL*_7**
nuoD	*nuoD*_4	*nuoD*_1	***nuoD*_4**	***nuoD*_4**	***nuoD*_4**	***nuoD*_4**
ppsA	*ppsA*_6	***ppsA*_6**	*ppsA*_1	*ppsA*_23	***ppsA*_6**	***ppsA*_6**
trpE	*trpE*_7	*trpE*_3	***trpE*_7**	*trpE*_1	***trpE*_7**	***trpE*_7**
Sequence type*	27	253	549	146	**27**	**27**

*as given by the MLST 2.0 software[1].

Table S2: Calculated average nucleotide identity (ANI) values for 104 *P. aeruginosa* strains (*P. a.*) and 8 other *Pseudomonas* strains vs. *P. aeruginosa* KRP1. Shown are ANI values of different *Pseudomonas* strains versus the *P. aeruginosa* KRP1 strain. Values are given in %. The higher the respective number, the greater is the nucleotide identity between the respective genomes. Values were calculated within the EDGAR platform[2,3]. The numbers in parentheses are the respective NCBI accession numbers.

**Strain**	**ANI [%]**	**Strain**	**ANI [%]**
P. a. PA7 (NC_009656.1)	92.70	*P. a.* H27930 (CP008860.2)	98.95
P. a. VRFPA04 (CP008739.2)	97.91	*P. a.* F63912 (CP008858.2)	98.95
P. a. M1608 (CP008862.2)	98.01	*P. a.* M8A4 (CP015648.1)	98.96
P. a. 12939 (CP024477.1)	98.07	*P. a.* 8380 (AP014839.2)	98.97
P. a. PB368 (CP025050.1)	98.15	*P. a.* PAER4_119 (CP013113.1)	98.97
P. a. NCGM257 (AP014651.1)	98.22	*P. a.* VA-134 (CP013245.1)	98.97
P. a. 39016 (CM001020.1)	98.23	*P. a.* PA96 (CP007224.1)	98.98
P. a. NCGM 1900 (AP014622.1)	98.23	*P. a.* M28A1 (CP015649.1)	98.98
P. a. Cu1510 (CP013144.1)	98.24	*P. a.* W36662 (CP008870.2)	98.99
P. a. Pa58 (CP021775.1)	98.27	*P. a.* Pa84 (CP021999.1)	98.99
P. a. NCGM2.S1 (NC_017549.1)	98.31	*P. a.* F30658 (CP008857.1)	99.00
P. a. PASGNDM345 (CP020703.1)	98.31	*P. a.* M8A1 (CP015647.1)	99.02
P. a. E6130952 (CP020603.1)	98.31	*P. a.* ATCC 27853 (CP015117.1)	99.02
P. a. IOMTU 133 (AP017302.1)	98.33	*P. a.* W16407 (CP008869.2)	99.02
P. a. B10W (CP017969.1)	98.35	*P. a.* Pa1207 (CP022001.1)	99.03
P. a. M37351 (CP008863.1)	98.35	*P. a.* C-NN2 (LT883143.1)	99.04
P. a. UCBPP-PA14 (NC_008463.1)	98.36	*P. a.* PB353 (CP025051.1)	99.04
P. a. PA14OR (LT608330.1)	98.36	*P. a.* RP73 (NC_021577.1)	99.07
P. a. PB350 (CP025055.2)	98.44	*P. a.* DK1 (LN870292.1)	99.08
P. a. PB367 (CP025056.1)	98.44	*P. a.* NHmuc (CP013479.1)	99.08
P. a. MTB-1 (NC_023019.1)	98.45	*P. a.* YL84 (CP007147.1)	99.08
P. a. DN1 (CP017099.1)	98.48	*P. a.* SCVJan (CP013478.1)	99.08
P. a. PA_D1 (CP012585.1)	98.52	*P. a.* SCV20265 (NC_023149.1)	99.09
P. a. Ocean-1155 (CP022526.1)	98.52	*P. a.* S86968 (CP008865.2)	99.11
P. a. B136-33 (NC_020912.1)	98.54	*P. a.* 12-4-4(59) (CP013696.1)	99.14
P. a. L10 (CP019338.1)	98.57	*P. a.* T52373 (CP008867.1)	99.15
P. a. Pa124 (CP021774.1)	98.58	*P. a.* Pb18 (CP015650.1)	99.16
P. a. PAK (CP020659.1)	98.59	*P. a.* C7447m (NC_022360.1)	99.17
P. a. Pa127 (CP022000.1)	98.59	*P. a.* T38079 (CP008866.2)	99.17
P. a. S04 90 (CP011369.1)	98.61	*P. a.* DSM 50071 (CP012001.1)	99.18
P. a. PA_154197 (CP014866.1)	98.62	*P. a.* M18 (NC_017548.1)	99.18
P. a. BAMCPA07-48 (CP015377.1)	98.63	*P. a.* NCTC10332 (LN831024.1)	99.18
P. a. LT969520 (LT969520.1)	98.72	*P. a.* F9670 (CP008873.1)	99.18
P. a. PA83 (CP017293.1)	98.72	*P. a.* PA_150577 (CP017306.1)	99.18
P. a. DK2 (NC_018080.1)	98.74	*P. a.* F22031 (CP007399.1)	99.23
P. a. PA11803 (CP015003.1)	98.74	*P. a.* PAO1 (NC_002516.2)	99.24
P. a. PA7790 (CP014999.1)	98.74	*P. a.* ATCC 15692 (CP017149.1)	99.24
P. a. PA8281 (CP015002.1)	98.75	*P. a.* PAO1H2O (CP008749.1)	99.25
P. a. Carb01 63 (CP011317.1)	98.76	*P. a.* SJTD-1 (CP015877.1)	99.25
P. a. CCBH4851 (CP021380.2)	98.78	*P. a.* W60856 (CP008864.2)	99.25
P. a. H47921 (CP008861.1)	98.78	*P. a.* PA121617 (CP016214.1)	99.25
P. a. Pa1242 (CP022002.1)	98.78	*P. a.* H5708 (CP008859.2)	99.26
P. a. RIVM-EMC2982 (CP016955.1)	98.78	*P. a.* USDA-ARS-USMARC-41639 (CP013989.1)	99.29
P. a. 19BR (AFXJ01000001.1)	98.80	*P. a.* X78812 (CP008872.2)	99.31
P. a. F23197 (CP008856.2)	98.80	*P. a.* F9676 (CP012066.1)	99.37
P. a. LESB58 (NC_011770.1)	98.81	*P. a.* W45909 (CP008871.2)	99.96
P. a. PA1088 (CP015001.1)	98.82	*P. a.* FA-HZ1 (CP017353.1)	99.98
P. a. PA38182 (HG530068.1)	98.84		
P. a. PA1 (NC_022808.2)	98.85		
P. a. PA1RG (CP012679.1)	98.85	*P. psychrotolerans* PRS08 (CP018758.1)	74.38
P. a. DHS01 (CP013993.1)	98.87	*P. putida* KT2440 (NC_002947.4)	75.41
P. a. N17-1 (CP014948.1)	98.89	*P. pseudoalcaligenes* KF707 (AJMR00000000.1)	77.94
P. a. PSE305 (HG974234.1)	98.90	*P. knackmussii* B13 (HG322950.1)	79.68
P. a. PACS2 (AAQW01000001.1)	98.92	*P. citronellolis* P3B5 (CP014158.1)	80.4
P. a. FRD1 (CP010555.1)	98.92	*P. stutzeri* A1501 (NC_009434.1)	75.83
P. a. PAO581 (NC_022361.1)	98.93	*P. pseudoalcaligenes* CECT5344 (HG916826.1)	76.79
P. a. T63266 (CP008868.1)	98.93	*P. fulva* 12-X (CP002727.1)	76.24

Table S3: Clusters of Orthologous Groups (COG) classification of singleton hits of *P. aeruginosa* KRP1. Singletons of *P. aeruginosa* KRP1 are denoted with their respective locus tag. The COG categories and the exact COG annotations are shown, for all singletons of KRP1 with respect to *P. aeruginosa* PAO1, PA14, LESB58, FA-HZ1 and W45909, which could be identified within the COG database. The percent identity of the respective singletons with the deposited database is also given.

KRP1 locus tag	COG annotation	identity [%]	COG functional category
KRP1_11685	Archaeal DNA helicase HerA or a related bacterial ATPase	64.64	[L] Replication and repair
KRP1_14955	Transposase InsO and inactivated derivatives	75	[X] Phage-derived proteins, transposases and other mobilome components
KRP1_23130	Molybdopterin or thiamine biosynthesis adenylyltransferase	85.45	[H] Coenzyme metabolism
KRP1_23140	Patatin-like phospholipase/acyl hydrolase	91.89	[R] General functional prediction only
KRP1_27620	phage Mu *gpF*-like domain	62.57	[S] Function Unknown
KRP1_27630	Phage terminase large subunit	61.65	[X] Phage-derived proteins, transposases and other mobilome components

Table S4: Absolute number of open reading frames (ORFs) belonging to different Clusters of Orthologous Groups (COG) database categories of areas I to V (Figure 4). Areas correspond to groups of genes, which are singletons to KRP1 or shared by KRP1 and up to five other *P. aeruginosa* strains; see Figure 4**.** Official COG categories are denoted and for each category the absolute number of genes for each area are given.

Clusters of Orthologous Groups (COGs) database categories	Area I (583)	Area II (463)	Area III (200)	Area IV (55)	Area V (102)
**Poorly characterized**					
No hit	439	330	22	40	78
**[R]** General function prediction only	10	13	22	5	4
**[S]** Function unknown	10	10	10	1	
**Information storage and processing**					
**[A]** RNA processing and modification			1		
**[J]** Translation, ribosomal structure and biogenesis	4	8	10		1
**[K]** Transcription	18	16	8		
**[L]** Replication, recombination and repair	19	10	4	1	
**Cellular processes and signaling**					
**[D]** Cell cycle control, cell division, chromosome partitioning	5	5	8		
**[M]** Cell wall/membrane/envelope biogenesis	12	10	16		2
**[N]** Cell motility	2	2	4		
**[O]** Post-translational modification, protein turnover, and chaperones	10	7	6		
**[T]** Signal transduction mechanisms	7	9	9	2	2
**[U]** Intracellular trafficking, secretion, and vesicular transport	10	5	9	1	8
**[V]** Defense mechanisms	3	2	9		
**[W]** Extracellular structures			1		
**Metabolism**					
**[C]** Energy production and conversion	3	5	32	1	
**[E]** Amino acid transport and metabolism	7	7	17		2
**[F]** Nucleotide transport and metabolism	2	3	4	1	
**[G]** Carbohydrate transport and metabolism	4	5	16		
**[H]** Coenzyme transport and metabolism	5	3	6		2
**[I]** Lipid transport and metabolism	5	8	13	2	2
**[P]** Inorganic ion transport and metabolism	18	14	12	1	
**[Q]** Secondary metabolites biosynthesis, transport, and catabolism	5	6	7	1	3
**[X]** Phage-derived proteins, transposases and other mobilome components	14	10		1	1

Table S5: PHASTER prediction for putative genomic islands (GIs) of KRP1 characterized as prophages and phage-like elements. The GI numbers given correspond to the GIs introduced in Table 2. For each prophages and phage-like elements the phage with the highest similarity is given, and the number of homologues ORFs, found in the PHASTER database is stated in parentheses. Additionally, the software provides a prediction if the phage is intact, questionable or incomplete, depending on the hit percent, also shown within the table.

GI	Most common phage (number of homologues ORFs**)	Completeness*	Hit [%]
GI 3	*Pseudomonas* phage YMC11/02/R656 [NC_028657] (9)	intact	71.9
PI 8	Bacteriophage N15 [NC_001901] (3)	questionable	26.8
PI 19a	*Pseudomonas* phage φCTX [NC_003278] (34)	intact	56.2
PI 19d	*Prochlorococcus* phage P-SSM3 [NC_021559] (1)	incomplete	12.8
GI 20a	*Mycobacterium* phage Catalina [NC_031238] (4)	incomplete	12.3
GI 23	*Pseudomonas* phage JBD93 [NC_030918] (30)	intact	96.2
PI 24d	*Pseudomonas* phage φCTX [NC_003278] (36)	intact	67.2

* based on PHASTER score
** CDS of GI found in PHASTER Database

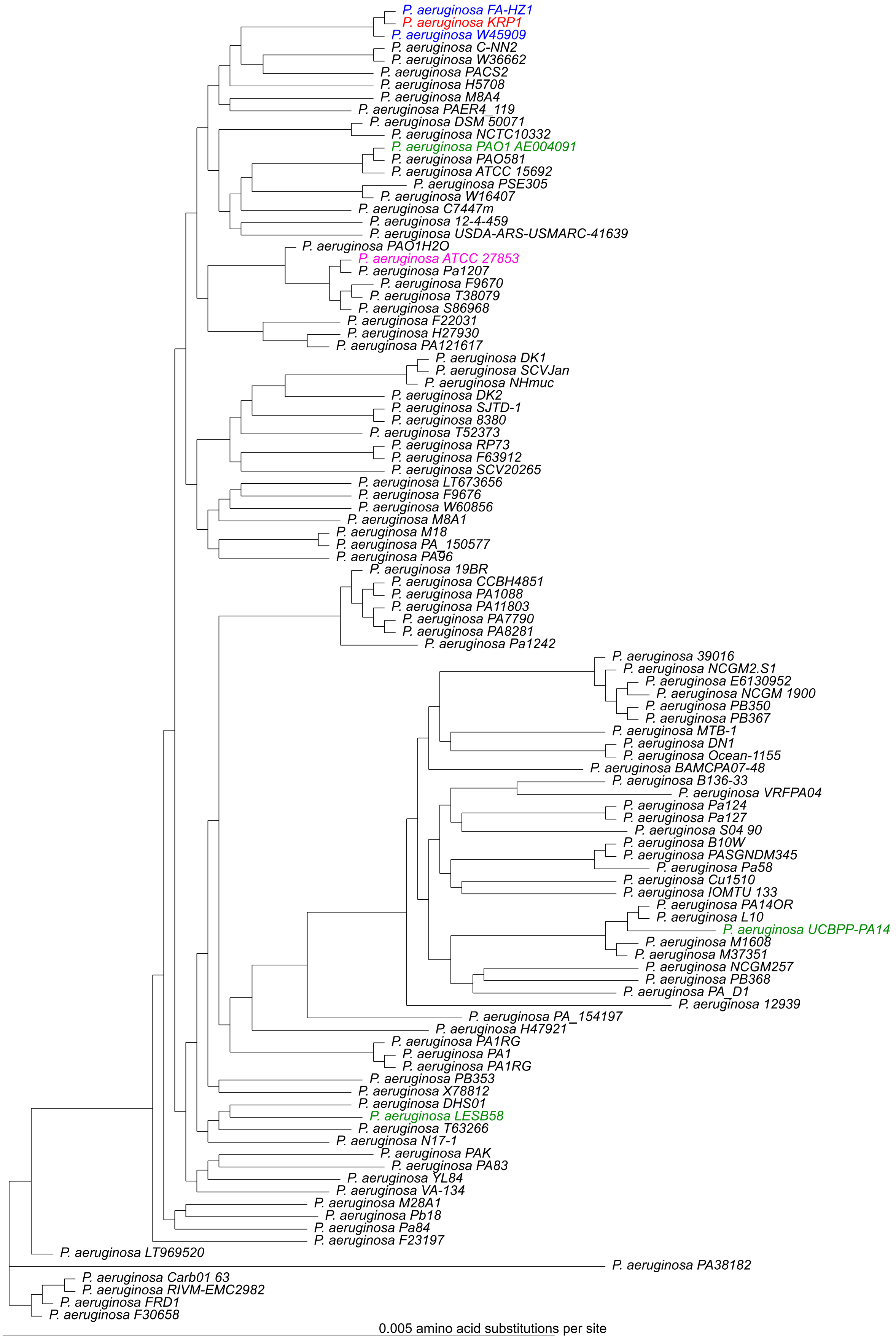
P. aeruginosa FA-HZ1
P. aeruginosa KRP1
P. aeruginosa W45909
P. aeruginosa C-NN2
P. aeruginosa W36662
P. aeruginosa PACS2
P. aeruginosa H5708
P. aeruginosa M8A4
P. aeruginosa PAER4_119
P. aeruginosa DSM 50071
P. aeruginosa NCTC10332
P. aeruginosa PAO1 AE004091
P. aeruginosa PAO581
P. aeruginosa ATCC 15692
P. aeruginosa PSE305
P. aeruginosa W16407
P. aeruginosa C7447m
P. aeruginosa 12-4-459
P. aeruginosa USDA-ARS-USMARC-41639
P. aeruginosa PAO1H2O
P. aeruginosa ATCC 27853
P. aeruginosa Pa1207
P. aeruginosa F9670
P. aeruginosa T38079
P. aeruginosa S86968
P. aeruginosa F22031
P. aeruginosa H27930
P. aeruginosa PA121617
P. aeruginosa DK1
P. aeruginosa SCVJan
P. aeruginosa NHmuc
P. aeruginosa DK2
P. aeruginosa SJTD-1
P. aeruginosa 8380
P. aeruginosa T52373
P. aeruginosa RP73
P. aeruginosa F63912
P. aeruginosa SCV20265
P. aeruginosa LT673656
P. aeruginosa F9676
P. aeruginosa W60856
P. aeruginosa M8A1
P. aeruginosa M18
P. aeruginosa PA_150577
P. aeruginosa PA96
P. aeruginosa 19BR
P. aeruginosa CCBH4851
P. aeruginosa PA1088
P. aeruginosa PA11803
P. aeruginosa PA7790
P. aeruginosa PA8281
P. aeruginosa Pa1242
P. aeruginosa 39016
P. aeruginosa NCGM2.S1
P. aeruginosa E6130952
P. aeruginosa NCGM 1900
P. aeruginosa PB350
P. aeruginosa PB367
P. aeruginosa MTB-1
P. aeruginosa DN1
P. aeruginosa Ocean-1155
P. aeruginosa BAMCPA07-48
P. aeruginosa B136-33
P. aeruginosa VRFPA04
P. aeruginosa Pa124
P. aeruginosa Pa127
P. aeruginosa S04 90
P. aeruginosa B10W
P. aeruginosa PASGNDM345
P. aeruginosa Pa58
P. aeruginosa Cu1510
P. aeruginosa IOMTU 133
P. aeruginosa PA14OR
P. aeruginosa L10
P. aeruginosa UCBPP-PA14
P. aeruginosa M1608
P. aeruginosa M37351
P. aeruginosa NCGM257
P. aeruginosa PB368
P. aeruginosa PA_D1
P. aeruginosa 12939
P. aeruginosa PA_154197
P. aeruginosa H47921
P. aeruginosa PA1RG
P. aeruginosa PA1
P. aeruginosa PA1RG
P. aeruginosa PB353
P. aeruginosa X78812
P. aeruginosa DHS01
P. aeruginosa LESB58
P. aeruginosa T63266
P. aeruginosa N17-1
P. aeruginosa PAK
P. aeruginosa PA83
P. aeruginosa YL84
P. aeruginosa VA-134
P. aeruginosa M28A1
P. aeruginosa Pb18
P. aeruginosa Pa84
P. aeruginosa F23197
P. aeruginosa LT969520
P. aeruginosa PA38182
P. aeruginosa Carb01 63
P. aeruginosa RIVM-EMC2982
P. aeruginosa FRD1
P. aeruginosa F30658
0.005 amino acid substitutions per site

Figure S1: Phylogenetic tree of 105 fully sequenced *P. aeruginosa* strains. *P. aeruginosa* KRP1 (red), the two closest relatives *P. aeruginosa* FA-HZ1 and W45909 (blue), the model strain PAO1, the common research strain PA14 and the potent virulent strain LESB58 (green) and the strain ATCC 27853 (pink), which showed the highest 16S rRNA similarity in the original publication[4], are highlighted. The tree was calculated using the EDGAR platform[2,3] out of a core of 2,567 genes per genome, which transcribe to 780,580 amino acid residues per genome.

References

1. Larsen, M. V. *et al.* Multilocus sequence typing of total-genome-sequenced bacteria. *J Clin Microbiol* **50**, 1355-1361, (2012).
2. Blom, J. *et al.* EDGAR: A software framework for the comparative analysis of prokaryotic genomes. *BMC Bioinf* **10**, 154, (2009).
3. Blom, J. *et al.* EDGAR 2.0: an enhanced software platform for comparative gene content analyses. *Nucleic Acids Res* **44**, W22-W28, (2016).
4. Rabaey, K., Boon, N., Siciliano, S. D., Verhaege, M. & Verstraete, W. Biofuel cells select for microbial consortia that self-mediate electron transfer. *Appl Environ Microbiol* **70**, 5373-5382, (2004).
